# Subtractive Cell-SELEX Selection of DNA Aptamers Binding Specifically and Selectively to Hepatocellular Carcinoma Cells with High Metastatic Potential 

**DOI:** 10.1155/2016/5735869

**Published:** 2016-03-28

**Authors:** Hao Chen, Chun-Hui Yuan, Yi-Fei Yang, Chang-Qing Yin, Qing Guan, Fu-Bing Wang, Jian-Cheng Tu

**Affiliations:** ^1^Department of Laboratory Medicine and Center for Gene Diagnosis, Zhongnan Hospital of Wuhan University, 169 Donghu Road, Wuchang District, Wuhan 430071, China; ^2^Department of Immunology, School of Basic Medical Sciences, Wuhan University, 185 Donghu Road, Wuchang District, Wuhan 430071, China

## Abstract

Relapse and metastasis are two key risk factors of hepatocellular carcinoma (HCC) prognosis; thus, it is emergent to develop an early and accurate detection method for prognostic evaluation of HCC after surgery. In this study, we sought to acquire oligonucleotide DNA aptamers that specifically bind to HCC cells with high metastatic potential. Two HCC cell lines derived from the same genetic background but with different metastatic potential were employed: MHCC97L (low metastatic properties) as subtractive targets and HCCLM9 (high metastatic properties) as screening targets. To mimic a fluid combining environment, initial DNA aptamers library was firstly labelled with magnetic nanoparticles using biotin-streptavidin system and then applied for aptamers selection. Through 10-round selection with subtractive Cell-SELEX, six aptamers, LY-1, LY-13, LY-46, LY-32, LY-27/45, and LY-7/43, display high affinity to HCCLM9 cells and do not bind to MHCC97L cells, as well as other tumor cell lines, including breast cancer, lung cancer, colon adenocarcinoma, gastric cancer, and cervical cancer, suggesting high specificity for HCCLM9 cells. Thus, the aptamers generated here will provide solid basis for identifying new diagnostic targets to detect HCC metastasis and also may provide valuable clues for developing new targeted therapeutics.

## 1. Introduction

Hepatocellular carcinoma (HCC) is the second most common cause of cancer-related death worldwide, estimated to be responsible for nearly 746,000 deaths in 2012 (9.1% of the total), and is a formidable public health challenge of China where 50% of the estimated 782,000 new cancer cases worldwide occurred [1, 2]. In recent decades, great advancements have been achieved in the development of therapeutics for HCC; besides hepatic resection as a mainstay of HCC treatment, local ablative therapies have greatly improved patient survival when HCC is diagnosed at early stages and, of them, radiofrequency ablation (RFA) is considered the reference standard [3–5]. However, according to the data presented by WHO in 2012 (http://globocan.iarc.fr/Default.aspx), the prognosis for hepatocellular carcinoma is still very poor (overall ratio of mortality to incidence is 0.95) [2, 6, 7].

As the two pivotal prognostic factors of HCC, postoperatively relapse and metastasis significantly shorted the survival time of surgically treated patients [8–10]. Currently, regular reexamination of serum alpha fetoprotein (AFP) level or contrast enhanced ultrasound (CEUS) still represents the two preferred diagnostic strategies in clinical examination to detect postoperatively relapse and metastasis [11]. However, with regard to early diagnosis of HCC, the positive rate of AFP is only 60–80% and often resulted in a false-positive result during pregnancy, as well as for active liver disease, embryonic tumor, and certain gastrointestinal tumors [12]. CEUS has been applied for more than ten years and has proved to be of great value in the management of HCC [13]. In most of the cases, HCC always shows earlier enhancement than the surrounding liver tissue; the detection rate in lesions larger than 2.1 cm is up to 92%–100% [14, 15]. However, when lesions are less than 1.0 cm, the detection rate is lower than 67%, and, apparently, CEUS has a relatively low ability to determine the smaller lesions of HCC in an early stage [16]. Thus, the identification of new tumor biomarkers involved in metastasis and recurrence is urgent in surveillance for HCC.

Since potential biomarkers can encompass various types of molecules ranging from glycolipids to proteins, thus, the strategy of Systematic Evolution of Ligands through Exponential Enrichment (SELEX) is ideally suited for the creation of biomarker, as aptamers generated by SELEX are capable of selective binding to any class of molecules [17]. Aptamers are synthetic, single-stranded oligonucleotides DNA or RNA that could fold into unique structures, including hairpin, fake festival, convex ring, and G-tetramer, to bind specifically to their target molecules [18]. Compared with antibodies, they possess several key advantages: smaller molecular weight (the average molecular weight of a DNA aptamer is about 25 kDa); without immunogenicity, greater specificity and affinity; and being easier to be economically produced and modified with multiple chemical molecules [18, 19]. Thus, aptamers have been widely used in cell imaging [20], clinical diagnosis, and targeted therapeutics [21–23].

Cell-SELEX derives from traditional SELEX process and uses whole living cells as target [24]. With the help of this technology, aptamers can be obtained even without prior knowledge of potential target molecules of cancer cells [25]. More importantly, Cell-SELEX-based selection of aptamers against cancer cells has been reported in different cancers, including leukemia, lung cancer, colon cancer, glioma, and ovarian cancer, as well as in HCC [25–28]. However, no information was given on the ability of aptamer to differentiate tumor cells with metastatic potential in HCC. In the present study, two HCC cell lines derived from the same genetic background but with different metastatic potential were employed: MHCC97L (low metastatic properties) as counterparts and HCCLM9 (high metastatic properties) as screening targets. Initial DNA aptamers library was labelled with magnetic nanoparticles and then applied for aptamers selection in a fluid compartment. Six aptamers selected by the Cell-SELEX display high affinity to HCCLM9 cells and do not bind to MHCC97L cells and other tumor cell lines, suggesting specificity for HCCLM9 cells. Thus, the aptamers generated here will provide solid basis for identifying new diagnostic targets to detect HCC metastasis.

## 2. Materials and Methods

### 2.1. Cell Lines and Reagents

MHCC97L cell and HCCLM9 cell were obtained from research center of Zhongnan Hospital, Wuhan University, as we previously described [29] and cultured in RPMI1640 (Gibco) containing 10% FBS (Gibco) and 100 units/mL penicillin-streptomycin (Beyotime, Shanghai, China). Other cell lines were maintained at our laboratory. Salmon sperm DNA, yeast tRNA, and BSA were purchased from Roche (F. Hoffmann-La Roche Ltd., USA). Streptavidin-coated magnetic nanoparticles M-280 (Dynabeads) were used for modifying biotin-labelled single-stranded DNA (ssDNA) aptamers pool.

### 2.2. Random DNA Library and Primers

An initial ssDNA aptamer library (5′-ATCCAGAGTGACGCAGCA-N40-TGGACACGGT GGCTTAGT-3′) consisting of 40-base randomized sequences was synthesized (Invitrogen, Shanghai, China), where N represents a randomized nucleotide of either A, G, C, or T. Forward primer 1 (5′-ATCCAGAGTGACGCAGCA-3′) and biotin-labelled reverse primer 2 (5′-ACTAA GCCACCGTGTCCA-3′) were used for PCR amplification of the DNA library or to separate the single-stranded DNA by streptavidin-coated magnetic particles. The FAM-labelled forward primer 1 (SBS Genetech Co., Ltd.) was used to monitor progress of selection by flow cytometry (Beckman Coulter, USA).

### 2.3. Subtractive Cell-SELEX Procedure

Subtractive Cell-SELEX procedure was performed as we described previously [29]. Briefly, initial ssDNA aptamers library (8 nmol) was first dissolved in 1 mL precooled binding buffer (PBS 1 M, MgCl_2_ 0.1 mg/mL, yeast tRNA 1 mg/mL, BSA 0.1 mg/mL, and Salmon sperm DNA 100 *μ*g/mL) and then incubated with adherent HCCLM9 cells for 1 h in an orbital shaker. After incubation, the cells were washed 3 times with binding buffer to remove unbound ssDNA aptamers. Adherent HCCLM9 cells were scraped off and resuspended in 500 *μ*L DNase-free deionized water. Cell suspension was then heated at 100°C for 5 min and centrifuged at 12000 g for 5 min. Supernatant containing eluted ssDNA aptamers was collected and then amplified by PCR using biotin-labelled primer. The biotin-labelled ssDNA aptamers were then incubated with streptavidin-coated magnetic nanoparticles (Dynabeads, M-280 Streptavidin, Invitrogen) and used for the next round of selection.

From the fourth round of selection, the selected ssDNA aptamers pool was firstly incubated with subtractive MHCC97L cells to perform subtractive selection and thus filtered out ssDNA aptamers that may bind to subtractive MHCC97L cells. The unbounded aptamers pool was specifically targeted to HCCLM9 cells and then incubated with HCCLM9 cells for positive selection. Furthermore, the target cell number and the concentration of ssDNA aptamers pool were gradually reduced with the selective round proceeding to 10 cycles.

### 2.4. The Binding Ability of Each Round of Enriched ssDNA Aptamers Pool

FAM-labelled ssDNA aptamers pools of 3, 5, 7, and 9 rounds were incubated with target cells HCCLM9 or subtractive cells MHCC97L (1 × 10^6^ for each) in 500 *μ*L binding buffer on ice for 30 min. Cells were washed twice after incubation and the fluorescence intensity was determined by flow cytometry. The FAM-labelled control aptamer NK8 (bound to* Mycobacterium tuberculosis*) was used as a negative control [29, 30].

### 2.5. Clones Selection and Sequencing

The ssDNA aptamers pool of 9 rounds was amplified by PCR to obtain double-stranded DNAs (dsDNAs) using unmodified primers and then cloned into a T vector (Invitrogen). The recombinant plasmid was then transformed into* E. coli* DH5*α* and randomly selected 50 clones using blue-white selection. The selected 50 clones were sequenced by Invitrogen Co., Ltd. (Shanghai, China) and designated as LY1 to LY50.

### 2.6. Sequence Alignments and Secondary Structures Analysis of Aptamers

DNAMAN software version 6.0 (Lynnon Biosoft, CA, USA) was used for sequence analysis and alignments. RNA structure (version 4.5, University of Rochester Medical Center) and MEME online analysis software version 4.10.2 (http://meme-suite.org/tools/meme) were used to estimate the secondary structures of sequenced aptamers.

### 2.7. Fluorescence Imaging of the Selected Aptamers Bound to HCCLM9 Cells

The FAM-labelled aptamers were synthesized by SBS Genetech Co., Ltd. (Shanghai, China). HCCLM9 cells were cultured in chamber slides overnight. The FAM-labelled individual aptamer was incubated with cell monolayer in chamber slides in binding buffer on 4°C for 30 min. After washing 3 times with PBS, the cells were imaged with Olympus BX51 fluorescence microscope (Olympus, Tokyo, Japan).

### 2.8. Determination of the Dissociation Constants (*K*_*d*_) of Individual ssDNA Aptamers

To determine the *K*_*d*_ value of the six selected aptamers, HCCLM9 cells (1 × 10^6^) were incubated with various concentrations of FAM-labelled aptamers in binding buffer at 4°C for 30 min and the fluorescence intensity was determined by flow cytometry. The mean fluorescence intensity (MFI) of negative control aptamer NK8 was subtracted from that of each aptamer with the target cells to determine the specific binding of each aptamer. Then, the equilibrium dissociation constants (*K*_*d*_) of each aptamer were determined by nonlinear regression for one-site binding according to the equation: *Y* = *B*_max_ × *X*/(*K*_*d*_ + *X*) using GraphPad Prism version 5.0 (GraphPad Software, Inc.).

### 2.9. Specificity Analysis of Selected Aptamers

To determine the cell specificity of the selected aptamers, human cancer cell lines including HCC cell lines MHCC97L, HepG2, and Huh-7, breast cancer cell line MDA-MB-231, lung cancer cell line H1299, colon adenocarcinoma cell line SW48, gastric cancer cell line MGC803, and cervical cancer cell line HeLa were used to test the specific binding affinity with fluorescent microcopy assay.

## 3. Results

### 3.1. Aptamers Selection and Binding Affinity Analysis

Subtractive Cell-SELEX was performed using HCC cell line HCCLM9 cells (with high metastatic properties) as the target and MHCC97L cells (low metastatic properties) as subtractive target, respectively, for the selection of metastatic-specific aptamers. HCCLM9 and MHCC97L were differentiated from MHCC97 cells as shown in our previous studies [29, 31]. In order to get aptamers with high specificity, 10 rounds of selection were performed. And with increasing rounds of enrichment, the number of target cells, ssDNA concentration, and the incubation time were gradually decreased, while the number of washing times was increased to reinforce the selective pressure. In the selection procedure, we amplified the aptamer pools of 2, 4, 6, 8, 9, and 10 cycles and analyzed them with agarose gel electrophoresis. With the selection cycles increased, the gray value of corresponding pool was gradually increased in the same PCR cycle and was the highest in 9 cycles. In 10 cycles of selection, the corresponding gray value was decreased compared with 9 cycles; thus, we speculated that aptamers of 9 cycles were optimal and included ssDNA aptamers that specifically bind to HCCLM9 cells. Simultaneously, we compared minimum PCR amplification cycles of each round pool; the minimum PCR amplification cycles in 9 rounds were 8, significantly lower than other rounds; these results further confirmed that 9 rounds of enrichment were optimal.

**Figure 1:**
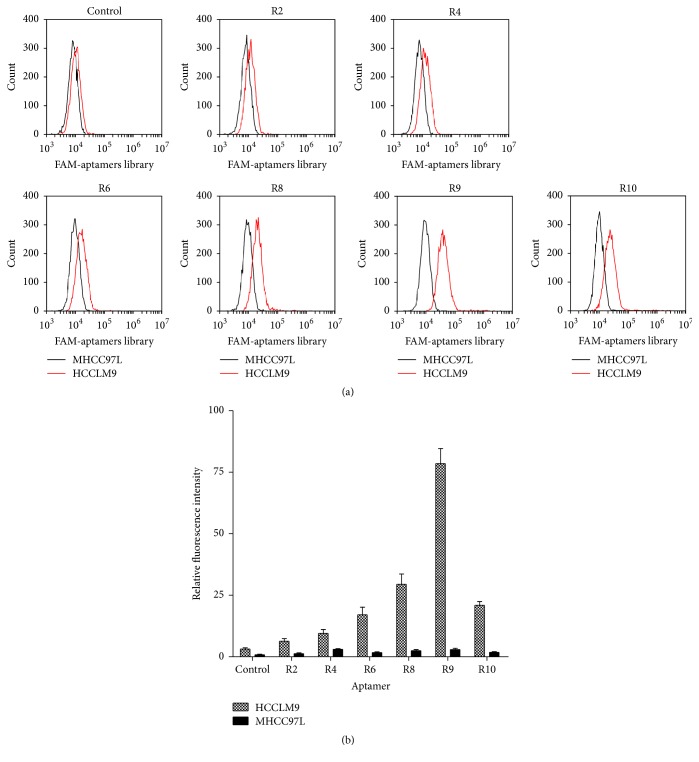
(a) The specific binding ability of aptamers library in selected rounds with HCCLM9 (target cells) and MHCC97L (subtractive cells) was analyzed by flow cytometry. With increasing rounds of enrichment, significant increases in fluorescence intensity were detected on HCCLM9 cells but not on MHCC97L and reached the peak at the ninth round. (b) Fluorescence shift was calculated using the equation (*F*_aptamer_ − *F*_cell_)/(*F*_library_ − *F*_cell_), where *F*_aptamer_, *F*_library_, and *F*_cell_ refer to the fluorescence of the selected aptamers pool, initial library, and the cell background, respectively.

Next, we labelled these selected rounds of aptamers with FAM and then incubated them with HCCLM9 cells and MHCC97L cells to examine the specific binding ability using flow cytometry. Relative fluorescence intensity was calculated as (*F*_aptamer_ − *F*_cell_)/(*F*_control_ − *F*_cell_). With increasing selective rounds, the relative fluorescence intensity of aptamer pool bound on HCCLM9 cells gradually increased and reached the highest value in 9 rounds, while the relative fluorescence intensity of MHCC97L cells showed no discernible change (Figure 1). Therefore, it was evident that the pool of ssDNA aptamers in 9 cycles has preferential and specific binding to HCCLM9 cells.

### 3.2. Selection, Sequencing, and Structure Prediction of Aptamers

**Figure 2:**
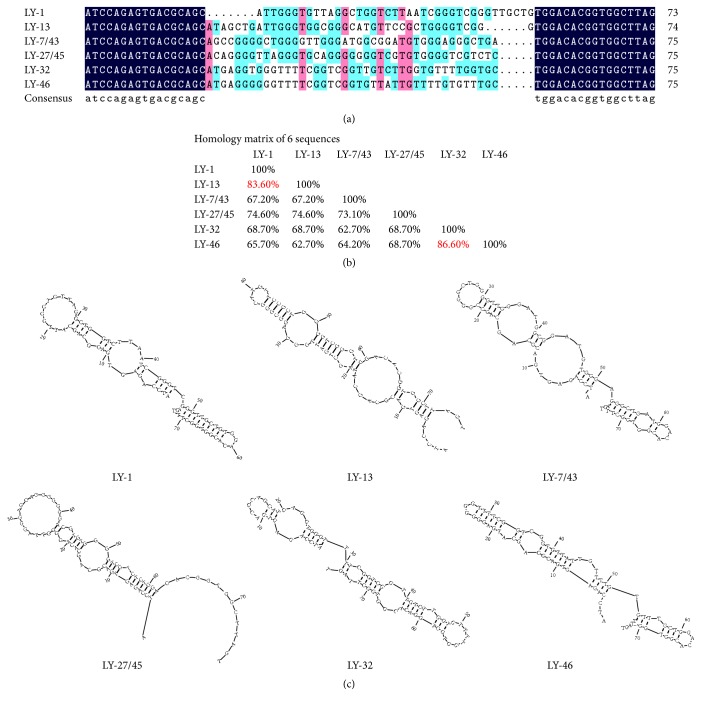
The homology analysis and secondary structure prediction of six aptamers. (a) Sequence alignment of six aptamers; all of the six aptamers showed a tendency towards G-richness, T-richness, or both. (b) Homology analysis of six aptamers; the greatest percentage of homology existed between LY-32 and LY-46; the second was LY-1 and LY-13. (c) Secondary structure prediction of six aptamers by RNA structure software.

Then, the ssDNA aptamers pool in 9 cycles was amplified into double strand and cloned into T vector and transformed into* E. coli* 5*α*. Next, fifty clones were randomly selected using blue-white selection and subjected to sequencing analysis. Among these 50 clones, 10 clones were discarded with no or multiple sequences detected. 23 clones yielding the same sequence are marked as LY-1, 9 clones yielding the same sequence are marked as LY-13, 2 clones yielding the same sequence are marked as LY-7/43, another 2 clones yielding the same sequence are marked as LY-27/45, and the rest 2 clones each yielding one sequence are marked as LY-32 and LY-46 (Figure 2(a)).

According to the sequencing result, we further analyzed the sequence homology and structure prediction of these six selected aptamers. The greatest percentage of homology existed between LY-32 and LY-46; the second was LY-1 and LY-13 (Figure 2(b)). Aptamers that are contained within homologous groups are likely to have been strongly preferentially enriched during the selection process. All of the six aptamers showed a tendency towards G-richness, T-richness, or both (Figure 2(a)). The secondary-structural analysis of the six chosen aptamers was shown in Figure 2(c).

### 3.3. Specific Binding Affinity of the Six Selected Aptamers

**Figure 3:**
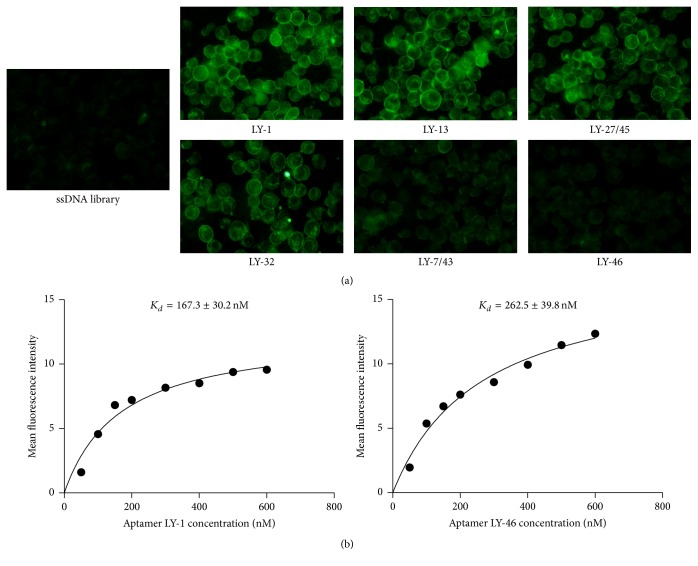
The specific binding affinity of these six selected aptamers to HCCLM9 cells. (a) The fluorescence imaging of six selected aptamers and initial ssDNA library control bound to HCCLM9 cells. Compared with the control group, all six selected aptamers were obviously bound to the membrane of target HCCLM9 cells; particularly, LY-1 and LY-13 had the highest binding capacity. (b) Binding curve of aptamers LY-1 and LY-46 with target HCCLM9 cells. Cells were incubated with increasing concentrations of FAM-labelled aptamers and the fluorescence intensity (MFI) was detected by flow cytometry. After subtracting the MFI of negative aptamer control (NK8), the MFI of FAM-aptamer bound target cells was used to calculate the *K*_*d*_ value of each aptamer.

To further confirm the specific binding affinity of these six selected aptamers to HCCLM9 cells, we firstly labelled aptamers with FAM at the 5′ end. And then their binding affinity to HCCLM9 cells was evaluated with fluorescent microcopy assay. The percentage of the cells with fluorescence above the set threshold was used to evaluate the binding capacity of the aptamer to the cells [32]. All six aptamers were significantly bound to the target HCCLM9 cells compared to the ssDNA library; particularly, LY-1 and LY-13 had the highest binding capacity (Figure 3(a)). Moreover, all six aptamers displayed high binding affinity to HCCLM9 cells with *K*_*d*_ values in the tiny range from 167.3 to 369.7 nM (Figure 3(b), Table 1), and LY-1 exhibited the highest binding affinity with a *K*_*d*_ value of 167.3 ± 30.2 nM.

Next, to test the binding specificity of these six aptamers, different HCC cells (MHCC97L, Huh-7, and HepG2), human lung cancer cell line H1299, human colon adenocarcinoma cell line SW48, human gastric cancer cell line MGC803, human cervical cancer cell line HeLa, human breast cancer cell line MDA-MB-231, and human peripheral WBC were used. As concluded in Table 2, all six aptamers exhibited specific binding affinity to HCCLM9 cells to different extent. LY-1 and LY-13 also recognized other HCC cell lines, including MHCC97L, Huh-7, and HepG2 cells, while the rest aptamers only recognized one kind of the other HCC cell lines, beside HCCLM9 cells, and control aptamer NK8 showed no detectable binding affinity to any kinds of cells. However, these six aptamers exhibited no detectable recognition of any other tumor cell lines and normal white blood cells (WBC), revealing that the six aptamers specifically targeted metastatic HCC cells with high affinity, especially LY-1 and LY-13.

## 4. Discussion

When developing new molecular probes for cancers, Cell-SELEX has advantages over traditional protein-SELEX, as it could be fulfilled just depending on expressive difference of cell surface molecules between target cells and control cells, even without the need to figure out the detailed molecular characteristics of target cells [32]. More importantly, surface molecules of target cells can maintain native folding and glycosylation states and an improved specificity can further be achieved by performing an additional subtractive step [25]. The target molecules of aptamers selected by subtractive Cell-SELEX may be the different compositions between tumor cells and normal counterparts [33, 34]. Thus, studies focused on these different compositions may provide valuable clues for the development of new tumor-specific biomarkers or therapeutic targets.

In recent years, subtractive Cell-SELEX has been widely applied in identifying new tumor biomarker. In gastric cancer, Cao et al. [35] successfully selected ssDNA aptamer cy-apt 20 by using human gastric cancer AGS cells as target cells and normal gastric epithelium GES-1 cells as subtractive cells. In clinical reexamination, cy-apt 20 further showed great diagnostic and therapeutic potential of gastric cancer. Shangguan et al. [36] developed 6 Cell-SELEX-generated aptamers against neoplastic cells; among these aptamers, sgd5 recognized only its target Toledo cells, while sgc3, sgd2, sgd3, sgc4, and sgc8, selected from a T-cell acute leukemia (T-ALL) cell line CCRF-CEM cells, identified all of the cultured T-ALL cell lines. Furthermore, sgc8, sgc3, and sgd3 showed good selectivity toward T-ALL cells and neoplastic cells in patient samples, while almost not binding to normal hematopoietic cells or lymphoma and myeloma cells. Importantly, by using differential Cell-SELEX, Cerchia et al. developed five aptamers (GL36, GL35, GL44, GL43, and GL21) which specifically distinguished U87MG glioma cells from the less malignant cell line T98G and other nonrelated cancer cell types [28].

Metastasis is the major obstacle that resulted in the failure treatment and recurrence of HCC; thus, to identify new tumor biomarkers with great potential to reflect HCC-related metastasis has been a hot research point in recent years [37–39]. Gkretsi and Bogdanos [8] firstly demonstrated that migfilin promoted HCC metastasis* in vitro* and may serve as a potential therapeutic target. Aptamer is a new kind of biomarkers, which has been widely used in diagnosis of multiple cancers [40–42]. Kashefi-Kheyrabadi et al. [43] and Sun et al. [44, 45] coupled aptamer TLS11a with electrochemical biosensor which effectively differentiated HCC HepG2 cells from lung cancer or prostate cancer cells and provided a new target for early diagnosis. While most aptamers used HepG2 cells as selective target and normal liver cells as subtractive cells, thus, these aptamers could be only used for early diagnosis and showed no help in indicating metastasis of HCC [25]. Herein, we used high metastatic HCCLM9 cells as the target cells and the less metastatic MHCC97L cells as the negative cells to perform subtractive Cell-SELEX under strict selection conditions and thereby generated a panel of six aptamers that can specifically bind to HCC cells with high metastatic potential. It is worth noting that HCCLM9 cell line was derived from MHCC97L cells but with high metastatic potential, the different compositions between these two cell lines may only relate to metastasis [46, 47], and, thus, aptamers generated from these two cell lines may specifically recognize metastatic-related surface molecules expressed on HCC cells and could be potential biomarkers to predict metastasis of HCC.

In aptamers selection procedure, we randomly selected 50 clones of the nine rounds of ssDNA aptamers pool. Aptamers sequencing and alignments analysis revealed that five mutual motifs existed in these selected aptamers and continuous G-rich motif abundantly existed in our six selected aptamers, which showed high binding affinity to HCCLM9 cells. This result suggested that aptamers in our initial ssDNA pool were specifically enriched by subtractive Cell-SELEX technique [48]. These six aptamers will further be subdivided into four families according to mutual motifs existing in secondary structure analysis, and continuous G-rich motif form stem-loops in these aptamers. Next, we designated these aptamers as LY-1, LY-13, LY-46, LY-32, LY-27/45, and LY-7/43 and further labelled these six aptamers with FAM to examine the specific binding ability of individual aptamer to less metastatic HCC cells (MHCC97L, HepG2, and Huh-7) or different tumor cell lines, including breast cancer, lung cancer, colon adenocarcinoma, gastric cancer, and cervical cancer. Though the *K*_*d*_ values of six selected aptamers may be higher than the aptamer reported by other researchers [32, 49], the six selected aptamers showed high specificity and sensitivity as potential molecular probes. And no matter whether aptamers are modified with fluorophore or magnetic nanoparticle, the specific binding ability of aptamers to high metastatic HCC cells was not changed; this may hint that these aptamers could be further conjugated with new types of luminescent or imaging materials and thus be developed into promising molecular probes for early prediction of HCC metastasis.

## Figures and Tables

**Table 1:** *K*_*d*_s of six selected aptamers of the HCCLM9 cells.

Aptamer	*K*_*d*_ value (nM)
LY-1	167.3 ± 30.2
LY-13	185.6 ± 28.3
LY-27/45	303.6 ± 34.5
LY-32	245.7 ± 44.4
LY-7/43	369.7 ± 46.3
LY-46	262.5 ± 39.8

**Table 2:** The binding specificity of six aptamers to different cell lines.

Cell lines	Aptamers						
	LY-1	LY-13	LY-27/45	LY-32	LY-7/43	LY-46	Control
HCCLM9	++++	++++	++	++	+	+	−
MHCC97L	+	+	−	+	−	−	−
Huh7	+	+	−	−	+	−	−
HepG2	+	+	+	−	−	+	−
H1299	−	−	−	−	−	−	−
SW480	−	−	−	−	−	−	−
MGC803	−	−	−	−	−	−	−
HeLa	−	−	−	−	−	−	−
MDA-MB-231	−	−	−	−	−	−	−
WBC	−	−	−	−	−	−	−
